# Supplementary material for: Knockout and re-expression system for mutant analysis in primary mouse T cells
Source: Front Immunol. 2026 Oct 13;17:1946861. doi: 10.3389/fimmu.2026.1946861 (PMC13710325; doi:10.3389/fimmu.2026.1946861)
Supplement: Supplementary file 1 [file Image7.pdf]

(from 1-642 bp)

mCD3g-GFP-T2A-PuroR (1953 bp)

```
ATGGAGCAAAGGAAGGGACTTGCAGGGCTGTTTCTTGTCATCAGTCTTCTCCAGGGCACCGTCGCACAGACCAATAAGGCCAAGAATCTGGTTCAGGTGGACGGGTC
TACCTCGTTTCCTTCCCTGAACGTCCCGACAAAGAACAGTAGTCAGAAGAGGTCCCGTGGCAGCGTGTCTGGTTATTCCGGTTCTTAGACCAAGTCCACCTGCCCAG
M E Q R K G L A G L F L V I S L L Q G T V A Q T N K A K N L V Q V D G S
mCD3gFF-eGFP-T2A-puro
murine CD3gamma
```

```
CAGGGGGGACGGTTCAGTTTTGCTTACATGCGGGCTTACTGACAAGACTATTAAGTGGCTGAAAGACGGTTCCATCATTAGCCCTTTGAATGCAACTAAAAACACTT
GTCCCCCCTGCCAAGTCAAAACGAATGTACGCCCGAATGACTGTTCTGATAATTCACCGACTTTCTGCCAAGGTAGTAATCGGGAAACTTACGTTGATTTTTGTGAA
R G D G S V L L T C G L T D K T I K W L K D G S I I S P L N A T K N T
mCD3gFF-eGFP-T2A-puro
murine CD3gamma
```

```
GGAACCTGGGAAATAACGCTAAAGACCCTCGGGGGACATATCAATGTCAAGGGGCTAAGGAAACTTCAAATCCCTTGCAGGTCTATTACCGAATGTGCGAAAATTGT
CCTTGGACCCTTTATTGCGATTTCTGGGAGCCCCCTGTATAGTTACAGTTCCCCGATTCCTTTGAAGTTTAGGGAACGTCCAGATAATGGCTTACACGCTTTTAACA
W N L G N N A K D P R G T Y Q C Q G A K E T S N P L Q V Y Y R M C E N C
mCD3gFF-eGFP-T2A-puro
murine CD3gamma
```

```
ATCGAGCTGAATATCGGGACAATCAGTGGTTTCATTTTTGCCGAGGTcATatcgATATTCTTCCTCGCCCTGGGTGTCTATCTTATTGCTGGACAGGACGGTGTCCG
TAGCTCGACTTATAGCCCTGTTAGTCACCAAAGTAAAAACGGCTCCAgTAtagcTATAAGAAGGAGCGGGACCCACAGATAGAATAACGACCTGTCCTGCCACAGGC
I E L N I G T I S G F I F A E V I S I F F L A L G V Y L I A G Q D G V R
mCD3gFF-eGFP-T2A-puro
murine CD3gamma
```

```
GCAAagcAGAGCCTCCGATAAGCAAACCCTTCTCCAAAACGAACAACTCTacCAACCCTTGAAAGATAGAGAATAcGATCAATaCTCTCACCTCCAAGGTAATCAGC
CGTTtcgTCTCGGAGGCTATTCGTTTGGGAAGAGGTTTTGCTTGTTGAGAtgGTTGGGAACTTTCTATCTCTTATgCTAGTTAtGAGAGTGGAGGTTCCATTAGTCG
Q S R A S D K Q T L L Q N E Q L Y Q P L K D R E Y D Q Y S H L Q G N Q
mCD3gFF-eGFP-T2A-puro
murine CD3gamma
```

```
TTAGAAAAAAAGGAggctcgagtGGAGGTGGCATGGTGAGCAAGGGCGAGGAGCTGTTCACCGGGGTGGTGCCCATCCTGGTCGAGCTGGACGGCGACGTAAACGGC
AATCTTTTTTTCCTccgagctcaCCTCCACCGTACCACTCGTTCCCGCTCCTCGACAAGTGGCCCCACCACGGGTAGGACCAGCTCGACCTGCCGCTGCATTTGCCG
L R K K G G S S G G G M V S K G E E L F T G V V P I L V E L D G D V N G
mCD3gFF-eGFP-T2A-puro
mu...a   linker   eGFP
```

mCD3g-GFP-T2A-PuroR (1953 bp) (from 643-1284 bp)

CACAAGTTCAGCGTGTCCGGCGAGGGCGAGGGCGATGCCACCTACGGCAAGCTGACCCTGAAGTTCATCTGCACCACCGGCAAGCTGCCCGTGCCCTGGCCCACCCT
GTGTTCAAGTCGCACAGGCCGCTCCCGCTCCCGCTACGGTGGATGCCGTTCGACTGGGACTTCAAGTAGACGTGGTGGCCGTTCGACGGGCACGGGACCGGGTGGGA
H K F S V S G E G E G D A T Y G K L T L K F I C T T G K L P V P W P T L
mCD3gFF-eGFP-T2A-puro
eGFP
650 660 670 680 690 700 710 720 730 740

CGTGACCACCCTGACCTACGGCGTGCAGTGCTTCAGCCGCTACCCCGACCACATGAAGCAGCACGACTTCTTCAAGTCCGCCATGCCCGAAGGCTACGTCCAGGAGC
GCACTGGTGGGACTGGATGCCGCACGTCACGAAGTCGGCGATGGGGCTGGTGTACTTCGTCGTGCTGAAGAAGTTCAGGCGGTACGGGCTTCCGATGCAGGTCCTCG
V T T L T Y G V Q C F S R Y P D H M K Q H D F F K S A M P E G Y V Q E
mCD3gFF-eGFP-T2A-puro
eGFP
750 760 770 780 790 800 810 820 830 840 850

GCACCATCTTCTTCAAGGACGACGGCAACTACAAGACCCGCGCCGAGGTGAAGTTCGAGGGCGACACCCTGGTGAACCGCATCGAGCTGAAGGGCATCGACTTCAAG
CGTGGTAGAAGAAGTTCCTGCTGCCGTTGATGTTCTGGGCGCGGCTCCACTTCAAGCTCCCGCTGTGGGACCACTTGGCGTAGCTCGACTTCCCGTAGCTGAAGTTC
R T I F F K D D G N Y K T R A E V K F E G D T L V N R I E L K G I D F K
mCD3gFF-eGFP-T2A-puro
eGFP
860 870 880 890 900 910 920 930 940 950 960

GAGGACGGCAACATCCTGGGGCACAAGCTGGAGTACAACTACAACAGCCACAACGTCTATATCATGGCCGACAAGCAGAAGAACGGCATCAAGGTGAACTTCAAGAT
CTCCTGCCGTTGTAGGACCCCGTGTTCGACCTCATGTTGATGTTGTCGGTGTTGCAGATATAGTACCGGCTGTTCGTCTTCTTGCCGTAGTTCCACTTGAAGTTCTA
E D G N I L G H K L E Y N Y N S H N V Y I M A D K Q K N G I K V N F K I
mCD3gFF-eGFP-T2A-puro
eGFP
970 980 990 1,000 1,010 1,020 1,030 1,040 1,050 1,060 1,070

CCGCCACAACATCGAGGACGGCAGCGTGCAGCTCGCCGACCACTACCAGCAGAACACCCCCATCGGCGACGGCCCCGTGCTGCTGCCCGACAACCACTACCTGAGCA
GGCGGTGTTGTAGCTCCTGCCGTCGCACGTCGAGCGGCTGGTGATGGTCGTCTTGTGGGGGTAGCCGCTGCCGGGGCACGACGACGGGCTGTTGGTGATGGACTCGT
R H N I E D G S V Q L A D H Y Q Q N T P I G D G P V L L P D N H Y L S
mCD3gFF-eGFP-T2A-puro
eGFP
1,080 1,090 1,100 1,110 1,120 1,130 1,140 1,150 1,160 1,170

CCCAGTCCGCCCTGAGCAAAGACCCCAACGAGAAGCGCGATCACATGGTCCTGCTGGAGTTCGTGACCGCCGCCGGGATCACTCTCGGCATGGACGAGCTGTACAAG
GGGTCAGGCGGGACTCGTTTCTGGGGTTGCTCTTCGCGCTAGTGTACCAGGACGACCTCAAGCACTGGCGGCGGCCCTAGTGAGAGCCGTACCTGCTCGACATGTTC
T Q S A L S K D P N E K R D H M V L L E F V T A A G I T L G M D E L Y K
mCD3gFF-eGFP-T2A-puro
eGFP
1,180 1,190 1,200 1,210 1,220 1,230 1,240 1,250 1,260 1,270 1,280

mCD3g-GFP-T2A-PuroR (1953 bp) (from 1285-1926 bp)

ggcggccgcggagggGAGGGCCGAGGCAGCCTGCTGACCTGCGGCGACGTGGAGGAGAACCCCGGgCCCATGACCGAGTATAAACCTACTGTGCGCCTGGCAACAAG
ccgccggcgcctcccCTCCCGGCTCCGTCGGACGACTGGACGCCGCTGCACCTCCTCTTGGGGCCcGGGTACTGGCTCATATTTGGATGACACGCGGACCGTTGTTC
G G R G G E G R G S L L T C G D V E E N P G P M T E Y K P T V R L A T R
mCD3gFF-eGFP-T2A-puro
linker T2A PuroR
1,290 1,300 1,310 1,320 1,330 1,340 1,350 1,360 1,370 1,380 1,390

GGATGACGTTCCCAGGGCCGTACGCACCCTCGCCGCCGCATTTGCCGACTATCCTGCAACCAGGCATACTGTAGATCCTGACAGGCACATCGAGAGGGTCACAGAAT
CCTACTGCAAGGGTCCCGGCATGCGTGGGAGCGGCGGCGTAAACGGCTGATAGGACGTTGGTCCGTATGACATCTAGGACTGTCCGTGTAGCTCTCCCAGTGTCTTA
D D V P R A V R T L A A A F A D Y P A T R H T V D P D R H I E R V T E
mCD3gFF-eGFP-T2A-puro
PuroR
1,400 1,410 1,420 1,430 1,440 1,450 1,460 1,470 1,480 1,490

TGCAGGAATTGTTCCTGACTAGAGTTGGTTTGGATATAGGAAAAGTATGGGTGGCTGACGACGGAGCAGCCGTCGCTGTATGGACCACACCTGAATCTGTAGAAGCC
ACGTCCTTAACAAGGACTGATCTCAACCAAACCTATATCCTTTTCATACCCACCGACTGCTGCCTCGTCGGCAGCGACATACCTGGTGTGGACTTAGACATCTTCGG
L Q E L F L T R V G L D I G K V W V A D D G A A V A V W T T P E S V E A
mCD3gFF-eGFP-T2A-puro
PuroR
1,500 1,510 1,520 1,530 1,540 1,550 1,560 1,570 1,580 1,590 1,600

GGTGCTGTCTTCGCTGAGATCGGTCCTAGAATGGCAGAGTTGTCtGGAAGCAGGCTCGCCGCTCAACAGCAAATGGAGGGCCTGTTGGCCCCCCATCGCCCAAAAGA
CCACGACAGAAGCGACTCTAGCCAGGATCTTACCGTCTCAACAGaCCTTCGTCCGAGCGGCGAGTTGTCGTTTACCTCCCGGACAACCGGGGGGTAGCGGGTTTTCT
G A V F A E I G P R M A E L S G S R L A A Q Q Q M E G L L A P H R P K E
mCD3gFF-eGFP-T2A-puro
PuroR
1,610 1,620 1,630 1,640 1,650 1,660 1,670 1,680 1,690 1,700 1,710

ACCCGCTTGGTTTCTGGCAACCGTGGGCGTGAGCCCCGACCATCAAGGCAAAGGGTTGGGCAGtGCTGTTGTCCTCCCTGGAGTGGAAGCTGCCGAACGCGCAGGGG
TGGGCGAACCAAAGACCGTTGGCACCCGCACTCGGGGCTGGTAGTTCCGTTTCCCAACCCGTCaCGACAACAGGAGGGACCTCACCTTCGACGGCTTGCGCGTCCCC
P A W F L A T V G V S P D H Q G K G L G S A V V L P G V E A A E R A G
mCD3gFF-eGFP-T2A-puro
PuroR
1,720 1,730 1,740 1,750 1,760 1,770 1,780 1,790 1,800 1,810

TTCCAGCTTTTCTTGAAACCAGTGCCCCACGGAACTTGCCCTTCTACGAGAGACTTGGCTTCACCGTCACCGCCGACGTCGAGGTTCCTGAGGGTCCCAGGACTTGG
AAGGTCGAAAAGAACTTTGGTCACGGGGTGCCTTGAACGGGAAGATGCTCTCTGAACCGAAGTGGCAGTGGCGGCTGCAGCTCCAAGGACTCCCAGGGTCCTGAACC
V P A F L E T S A P R N L P F Y E R L G F T V T A D V E V P E G P R T W
mCD3gFF-eGFP-T2A-puro
PuroR
1,820 1,830 1,840 1,850 1,860 1,870 1,880 1,890 1,900 1,910 1,920

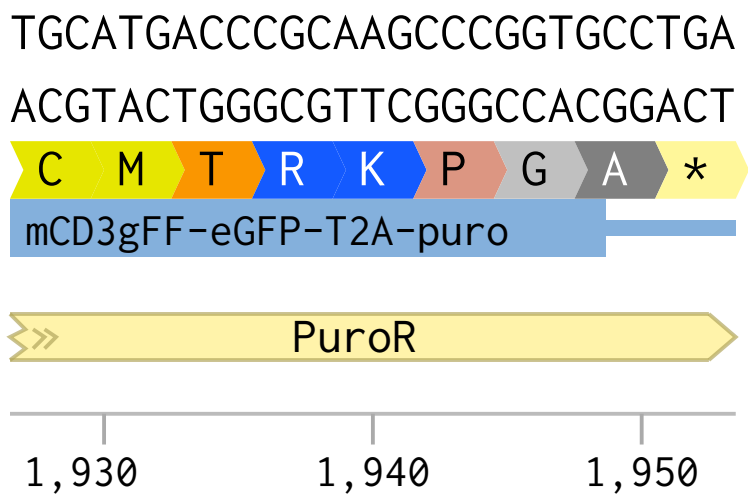
TGCATGACCCGCAAGCCCGGTGCCTGA
ACGTACTGGGCGTTCGGGCCACGGACT
C M T R K P G A *
mCD3gFF-eGFP-T2A-puro
PuroR
1,930
1,940
1,950
