# Supplementary material for: Knockout and re-expression system for mutant analysis in primary mouse T cells
Source: Front Immunol. 2026 Oct 13;17:1946861. doi: 10.3389/fimmu.2026.1946861 (PMC13710325; doi:10.3389/fimmu.2026.1946861)
Supplement: Supplementary file 2 [file Image8.pdf]

(from 1-415 bp)

mZap70WT-3xHAtag-T2A-PuroR (2631 bp)

```
ATGCCtGACCCcGCCGCACATtTGCCaTTtTTCTACGGCTCTATATCCCGCGCCGAAGCAGAGGAGCATCTGAAGcTGGCtGG
TACGGaCTGGGgCGGCGTGTAaACGGtAAaAAGATGCCGAGATATAGGGCGCGGCTTCGTCTCCTCGTAGACTTCgACCGaCC
M P D P A A H L P F F Y G S I S R A E A E E H L K L A G
1-1854
SH2_1 domain
mZap70 WT
10 20 30 40 50 60 70 80
```

```
CATGGCaGACGGTCTGTTcCTGCTCAGACAGTGCCTGAGATCaCTGGGTGGCTATGTACTGTCtCTcGTtCACGATGTgCGCT
GTACCGtCTGCCAGACAAgGACGAGTCTGTCACGGACTCTAGtGACCCACCGATACATGACAGaGAgCAaGTGCTACAcGCGA
M A D G L F L L R Q C L R S L G G Y V L S L V H D V R
1-1854
SH2_1 domain
mZap70 WT
90 100 110 120 130 140 150 160
```

```
TTCATCACTTTCCCATCGAACGGCAACTtAACGGTACATACGCaATCGCTGGTGGAAAAGCcCACTGTGGTCCCGCAGAACTC
AAGTAGTGAAAGGGTAGCTTGCCGTTGAaTTGCCATGTATGCGtTAGCGACCACCTTTTCGgGTGACACCAGGGCGTCTTGAG
F H H F P I E R Q L N G T Y A I A G G K A H C G P A E L
1-1854
SH2_1 domain
mZap70 WT
170 180 190 200 210 220 230 240
```

```
TGTCAGTTTTACAGTCAAGACCCCGATGGACTcCCTTGTAACCTtCGGAAACCATGCAATCGGCCACCCGGCCTCGAACCACA
ACAGTCAAAATGTCAGTTCTGGGGCTACCTGAgGGAACATTGGAaGCCTTTGGTACGTTAGCCGGTGGGCCGGAGCTTGGTGT
C Q F Y S Q D P D G L P C N L R K P C N R P P G L E P Q
1-1854
SH2_1 domain    Interdomain A
mZap70 WT
250 260 270 280 290 300 310 320 330
```

```
ACCTGGAGTCTTTGAtTGCTTGAGGGAcGCtATGGTTAGGGATTACGTCAGACAGACaTGGAAACTGGAAGGGGACGCCCTTG
TGGACCTCAGAAACTaACGAACTCCCTgCGaTACCAATCCCTAATGCAGTCTGTCTGtACCTTTGACCTTCCCCTGCGGGAAC
P G V F D C L R D A M V R D Y V R Q T W K L E G D A L
1-1854
Interdomain A
mZap70 WT
340 350 360 370 380 390 400 410
```

mZap70WT-3xHAtag-T2A-PuroR (2631 bp) (from 416-830 bp)

```
AGCAAGCcATTATAagtCAGGCCCCCCAGGTAGAGAAGCTCATCGCAACcACAGCTCACGAACGGATGCCTTGGTATCACTCC
TCGTTCGgTAATATtcaGTCCGGGGGGTCCATCTCTTCGAGTAGCGTTGgTGTCGAGTGCTTGCCTACGGAACCATAGTGAGG
E  Q  A  I  I  S  Q  A  P  Q  V  E  K  L  I  A  T  T  A  H  E  R  M  P  W  Y  H  S
1-1854
Interdomain A                                                          SH2_...main
mZap70 WT
420       430       440       450       460       470       480       490
```

```
tcaCTCACcaGaGAAGAGGCTGAGAGAAAgCTCTATTCAGGGCAACAGACtGACGGaAAaTTCtTgCTGcGgCCACGAAAGGA
agtGAGTGgtCtCTTCTCCGACTCTCTTTcGAGATAAGTCCCGTTGTCTGaCTGCCtTTtAAGaAcGACgCcGGTGCTTTCCT
S  L  T  R  E  E  A  E  R  K  L  Y  S  G  Q  Q  T  D  G  K  F  L  L  R  P  R  K  E
1-1854
SH2_2 domain
mZap70 WT
500       510       520       530       540       550       560       570       580
```

```
ACAAGGgACCTACGCcCTGTCCCTTGTTTATGGCAAGACCGTTTATCACTACcTcATcAGCCAAGACAAGGCtGGtAAaTAcT
TGTTCCcTGGATGCGgGACAGGGAACAAATACCGTTCTGGCAAATAGTGATGgAgTAgTCGGTTCTGTTCCGaCCaTTtATgA
Q  G  T  Y  A  L  S  L  V  Y  G  K  T  V  Y  H  Y  L  I  S  Q  D  K  A  G  K  Y
1-1854
SH2_2 domain
mZap70 WT
590       600       610       620       630       640       650       660
```

```
GTATtCCCGAAGGCACaAAgTTTGATACcCTCTGGCAACTgGTGGAATACCTcAAACTGAAAGCTGACGGcCTGATTTAtcGA
CATAaGGGCTTCCGTGtTTcAAACTATGgGAGACCGTTGAcCACCTTATGGAgTTTGACTTTCGACTGCCgGACTAAATagCT
C  I  P  E  G  T  K  F  D  T  L  W  Q  L  V  E  Y  L  K  L  K  A  D  G  L  I  Y  R
1-1854
SH2_2 domain
mZap70 WT
670       680       690       700       710       720       730       740
```

```
TTGAAGGAGGTGTGCCCTAAcAGTAGCGCCAGCGCAGCCGTAGCCGCACCAACCTTGCCAGCTCACCCATCAACtTTCACaCA
AACTTCCTCCACACGGGATTgTCATCGCGGTCGCGTCGGCATCGGCGTGGTTGGAACGGTCGAGTGGGTAGTTGaAAGTGtGT
L  K  E  V  C  P  N  S  S  A  S  A  A  V  A  A  P  T  L  P  A  H  P  S  T  F  T  Q
1-1854
SH2_2 domain          Interdomain B
mZap70 WT
750       760       770       780       790       800       810       820       830
```

```
GCCTCAAaGaCGGGTGGATACACTgAAtTCCGACGGGTAcACACCCGAGCCTGCAAGACTGGCCTCCAGCACTGATAAGCCCA
CGGAGTTtCtGCCCACCTATGTGAcTTaAGGCTGCCCATgTGTGGGCTCGGACGTTCTGACCGGAGGTCGTGACTATTCGGGT
  P  Q  R  R  V  D  T  L  N  S  D  G  Y  T  P  E  P  A  R  L  A  S  S  T  D  K  P
1-1854
Interdomain B
mZap70 WT
840  850  860  870  880  890  900  910

GACCCATGCCAATGGACACATCTGTTTATGAATCACCTTAtTCAGATCCCGAGGAGCTGAAGGATAAAAAATTGTTCCTTAAA
CTGGGTACGGTTACCTGTGTAGACAAATACTTAGTGGAATaAGTCTAGGGCTCCTCGACTTCCTATTTTTTAACAAGGAATTT
 R  P  M  P  M  D  T  S  V  Y  E  S  P  Y  S  D  P  E  E  L  K  D  K  K  L  F  L  K
1-1854
Interdomain B
mZap70 WT
920  930  940  950  960  970  980  990

CGAGAGAATTTGCTTGTGGCCGATATAGAACTTGGCTGCGGTAACTTTGGGTCAGTTCGCCAGGGGGTTTACCGAATGCGGAA
GCTCTCTTAAACGAACACCGGCTATATCTTGAACCGACGCCATTGAAACCCAGTCAAGCGGTCCCCCAAATGGCTTACGCCTT
 R  E  N  L  L  V  A  D  I  E  L  G  C  G  N  F  G  S  V  R  Q  G  V  Y  R  M  R  K
1-1854
Inte...in B  Protein Kinase domain
mZap70 WT
1,000  1,010  1,020  1,030  1,040  1,050  1,060  1,070

GAAACAAATCGACGTTGCTATTAAGGTACTTAAACAgGGCACCGAGAAAGCcGATAAAGACGAAATGATGCGAGAGGCtCAgA
CTTTGTTTAGCTGCAACGATAATTCCATGAATTTGTcCCGTGGCTCTTTCGgCTATTTCTGCTTTACTACGCTCTCCGaGTcT
  K  Q  I  D  V  A  I  K  V  L  K  Q  G  T  E  K  A  D  K  D  E  M  M  R  E  A  Q
1-1854
Protein Kinase domain
mZap70 WT
1,080  1,090  1,100  1,110  1,120  1,130  1,140  1,150  1,160

TTATGCAtCAACTGGATAACCCATACATTGTTCGGCTTATCGGTGTATGTCAAGCcGAGGCtCTCATGCTGGTAATGGAGATG
AATACGTaGTTGACCTATTGGGTATGTAACAAGCCGAATAGCCACATACAGTTCGgCTCCGaGAGTACGACCATTACCTCTAC
 I  M  H  Q  L  D  N  P  Y  I  V  R  L  I  G  V  C  Q  A  E  A  L  M  L  V  M  E  M
1-1854
Protein Kinase domain
mZap70 WT
1,170  1,180  1,190  1,200  1,210  1,220  1,230  1,240
```

mZap70WT-3xHAtag-T2A-PuroR (2631 bp) (from 1246-1660 bp)

GCAGGAGGCGGTCCCCTTCACAAATTTCTGCTTGGGAAGAAGGAAGAGATACCCGTGAGCAAcGTGGCtGAACTTTTGCAcCA
CGTCCTCCGCCAGGGGAAGTGTTTAAAGACGAACCCTTCTTCCTTCTCTATGGGCACTCGTTgCACCGaCTTGAAAACGTgGT
A G G G P L H K F L L G K K E E I P V S N V A E L L H Q
1-1854
Protein Kinase domain
mZap70 WT
1,250 1,260 1,270 1,280 1,290 1,300 1,310 1,320

AGTcGCgATGGGTATGAAATATTTGGAGGAAAAGAATTTCGTgCAtCGGGATCTGGCTGCACGCAATGTGTTGCTTGTGAAtC
TCAgCGcTACCCATACTTTATAAACCTCCTTTTCTTAAAGCAcGTaGCCCTAGACCGACGTGCGTTACACAACGAACACTTaG
V A M G M K Y L E E K N F V H R D L A A R N V L L V N
1-1854
Protein Kinase domain
mZap70 WT
1,330 1,340 1,350 1,360 1,370 1,380 1,390 1,400 1,410

GCCATTACGCAAAgATtAGCGAcTTCGGCCTgAGCAAAGCTCTGGGGGCCGACGAtAGCTATTATACTGCACGATCCGCAGGA
CGGTAATGCGTTTcTAaTCGCTgAAGCCGGAcTCGTTTCGAGACCCCCGGCTGCTaTCGATAATATGACGTGCTAGGCGTCCT
R H Y A K I S D F G L S K A L G A D D S Y Y T A R S A G
1-1854
Protein Kinase domain
mZap70 WT
1,420 1,430 1,440 1,450 1,460 1,470 1,480 1,490

AAATGGCCTCTtAAaTGGTACGCACCTGAATGTATcAATTTCCGAAAGTTTTCATCAAGGTCTGACGTaTGGAGTTACGGGGT
TTTACCGGAGAaTTtACCATGCGTGGACTTACATAgTTAAAGGCTTTCAAAAGTAGTTCCAGACTGCAtACCTCAATGCCCCA
K W P L K W Y A P E C I N F R K F S S R S D V W S Y G V
1-1854
Protein Kinase domain
mZap70 WT
1,500 1,510 1,520 1,530 1,540 1,550 1,560 1,570

gACTATGTGGGAGGCTTTTAGCTACGGTCAAAAACCTTACAAGAAGATGAAGGGGCCTGAGGTGTTGGACTTTATTAAACAAG
cTGATACACCCTCCGAAAATCGATGCCAGTTTTTGGAATGTTCTTCTACTTCCCCGGACTCCACAACCTGAAATAATTTGTTC
T M W E A F S Y G Q K P Y K K M K G P E V L D F I K Q
1-1854
Protein Kinase domain
mZap70 WT
1,580 1,590 1,600 1,610 1,620 1,630 1,640 1,650 1,660

mZap70WT-3xHAtag-T2A-PuroR (2631 bp) (from 1661-2075 bp)

GAAAACGAATGGAGTGTCCCCCAGAATGcCCCCCTGAGATGTACGCTCTGATGagcGACTGtTGGATATATAAGTGGGAaGAc
CTTTTGCTTACCTCACAGGGGGTCTTACgGGGGGACTCTACATGCGAGACTACtcgCTGACaACCTATATATTCACCCTtCTg
G K R M E C P P E C P P E M Y A L M S D C W I Y K W E D
1-1854
Protein Kinase domain
mZap70 WT
1,670 1,680 1,690 1,700 1,710 1,720 1,730 1,740

cGcCCTGAtTTTTTGACTGTAGAgCAGaGGATGaGaAATTACTATTATAGCCTcGCCtctCGaGCCGAAGGGCCACCCCAATG
gCgGGACTaAAAAACTGACATCTcGTCtCCTACtCtTTAATGATAATATCGGAgCGGagaGCtCGGCTTCCCGGTGGGGTTAC
R P D F L T V E Q R M R N Y Y Y S L A S R A E G P P Q C
1-1854
Protein Kinase domain
mZap70 WT
1,750 1,760 1,770 1,780 1,790 1,800 1,810 1,820

tGAgCAAGTGGCCGAaGCTGCaTGcGGAggttcaggaggcTACCCATACGACGTACCAGATTACGCTGGTGGCGGCTATCCGT
aCTcGTTCACCGGCTtCGACGtACgCCTccaagtcctccgATGGGTATGCTGCATGGTCTAATGCGACCACCGCCGATAGGCA
E Q V A E A A C G G S G G Y P Y D V P D Y A G G G Y P
1-1854 1855-2028
mZap70 WT linker 3xHAtag (YPYDVPDYA)
1,830 1,840 1,850 1,860 1,870 1,880 1,890 1,900

ATGATGTCCCGGACTATGCGGGAGgTGgaTACCCCTACGACGTGCCCGACTACGCCggttctggcGAGGGCCGAGGCAGCCTG
TACTACAGGGCCTGATACGCCCTCcACctATGGGGATGCTGCACGGGCTGATGCGGccaagaccgCTCCCGGCTCCGTCGGAC
Y D V P D Y A G G G Y P Y D V P D Y A G S G E G R G S L
1855-2028
3xHAtag (YPYDVPDYA) linker T2A peptide
1,910 1,920 1,930 1,940 1,950 1,960 1,970 1,980 1,990

CTGACCTGCGGCGACGTGGAGGAGAACCCCGGCCCCATGGGCACCGAGTATAAACCTACTGTGCGCCTGGCAACAAGGGATGA
GACTGGACGCCGCTGCACCTCCTCTTGGGGCCGGGGTACCCGTGGCTCATATTTGGATGACACGCGGACCGTTGTTCCCTACT
L T C G D V E E N P G P M G T E Y K P T V R L A T R D D
1855-2028 2029-2631
T2A peptide PuroR
2,000 2,010 2,020 2,030 2,040 2,050 2,060 2,070

CGTTCCCAGGGCCGTACGCACCCTCGCCGCCGCATTTGCCGACTATCCTGCAACCAGGCATACTGTAGATCCTGACAGGCACA
GCAAGGGTCCCGGCATGCGTGGGAGCGGCGGCGTAAACGGCTGATAGGACGTTGGTCCGTATGACATCTAGGACTGTCCGTGT
V P R A V R T L A A A F A D Y P A T R H T V D P D R H
2029-2631
PuroR
2,080 2,090 2,100 2,110 2,120 2,130 2,140 2,150

TCGAGAGGGTCACAGAATTGCAGGAATTGTTCCTGACTAGAGTTGGTTTGGATATAGGAAAAGTATGGGTGGCTGACGACGGA
AGCTCTCCCAGTGTCTTAACGTCCTTAACAAGGACTGATCTCAACCAAACCTATATCCTTTTCATACCCACCGACTGCTGCCT
I E R V T E L Q E L F L T R V G L D I G K V W V A D D G
2029-2631
PuroR
2,160 2,170 2,180 2,190 2,200 2,210 2,220 2,230 2,240

GCAGCCGTCGCTGTATGGACCACACCTGAATCTGTAGAAGCCGGTGCTGTCTTCGCTGAGATCGGTCCTAGAATGGCAGAGTT
CGTCGGCAGCGACATACCTGGTGTGGACTTAGACATCTTCGGCCACGACAGAAGCGACTCTAGCCAGGATCTTACCGTCTCAA
A A V A V W T T P E S V E A G A V F A E I G P R M A E L
2029-2631
PuroR
2,250 2,260 2,270 2,280 2,290 2,300 2,310 2,320

GTCtGGAAGCAGGCTCGCCGCTCAACAGCAAATGGAGGGCCTGTTGGCCCCCCATCGCCCAAAAGAACCCGCTTGGTTTCTGG
CAGaCCTTCGTCCGAGCGGCGAGTTGTCGTTTACCTCCCGGACAACCGGGGGGTAGCGGGTTTTCTTGGGCGAACCAAAGACC
S G S R L A A Q Q Q M E G L L A P H R P K E P A W F L
2029-2631
PuroR
2,330 2,340 2,350 2,360 2,370 2,380 2,390 2,400

CAACCGTGGGCGTGAGCCCCGACCATCAAGGCAAAGGGTTGGGCAGtGCTGTTGTCCTCCCTGGAGTGGAAGCTGCCGAACGC
GTTGGCACCCGCACTCGGGGCTGGTAGTTCCGTTTCCCAACCCGTCaCGACAACAGGAGGGACCTCACCTTCGACGGCTTGCG
A T V G V S P D H Q G K G L G S A V V L P G V E A A E R
2029-2631
PuroR
2,410 2,420 2,430 2,440 2,450 2,460 2,470 2,480 2,490

```
GCAGGGGTTCCAGCTTTTCTTGAAACCAGTGCCCCACGGAACTTGCCCTTCTACGAGAGACTTGGCTTCACCGTCACCGCCGA
CGTCCCCAAGGTCGAAAAGAACTTTGGTCACGGGGTGCCTTGAACGGGAAGATGCTCTCTGAACCGAAGTGGCAGTGGCGGCT
 A  G  V  P  A  F  L  E  T  S  A  P  R  N  L  P  F  Y  E  R  L  G  F  T  V  T  A  D
2029-2631
PuroR
    2,500     2,510     2,520     2,530     2,540     2,550     2,560     2,570
```

```
CGTCGAGGTTCCTGAGGGTCCCAGGACTTGGTGCATGACCCGCAAGCCCGGTGCCTGA
GCAGCTCCAAGGACTCCCAGGGTCCTGAACCACGTACTGGGCGTTCGGGCCACGGACT
  V  E  V  P  E  G  P  R  T  W  C  M  T  R  K  P  G  A  *
2029-2631
PuroR
  2,580     2,590     2,600     2,610     2,620     2,630
```
